# Social stigma, genetic services, and South Africa: is testing taboo?

**DOI:** 10.1007/s12687-026-00893-9

**Published:** 2026-05-08

**Authors:** Caitlin Ching Sent, Brendon Pearce

**Affiliations:** Genetics Department, Faculty of Agriscience, Stellenbosch University, Van Der Bijl Street, Stellenbosch, 7600 South Africa

**Keywords:** Stigma, South Africa, Non-communicable diseases, Genomic testing, Economic inequality

## Abstract

Genetic testing and counselling are invaluable tools for preventative and personalised care. Unfortunately, utilisation of these services in South Africa, and continental Africa more broadly, has been limited. South Africa, which suffers from a unique epidemiological state, offers these genetic services, but accessibility is limited, as is education around this topic. Furthermore, coupled with the previous limitations, there is a prevalent distrust of the healthcare system and the stakeholders involved therein. This is largely due to the sociopolitical history of the country, which has resulted in severe economic inequalities and racial marginalisation of majority population groups. To effectively reduce the disease burden that South Africa is currently experiencing, it is necessary to increase the accessibility and uptake of these services, particularly genetic testing. While geographic and financial barriers could be tackled governmentally, other barriers, such as stigma towards genetic testing, Westernised medicine in general, and those that provide it, need to be investigated before investing valuable resources into infrastructure. Understanding these barriers would provide critical insight into the demand for genetic testing and how uptake and motivation could be improved. This narrative review discusses potential barriers to uptake and consumer demand in South Africa, and concludes by tying all identified barriers into an overarching concern regarding stigma (particularly intersectional stigma) related to genetic testing in a South African context. By doing so, we identify several key information gaps that future research could fill.

## Introduction

**Fig. 1:**
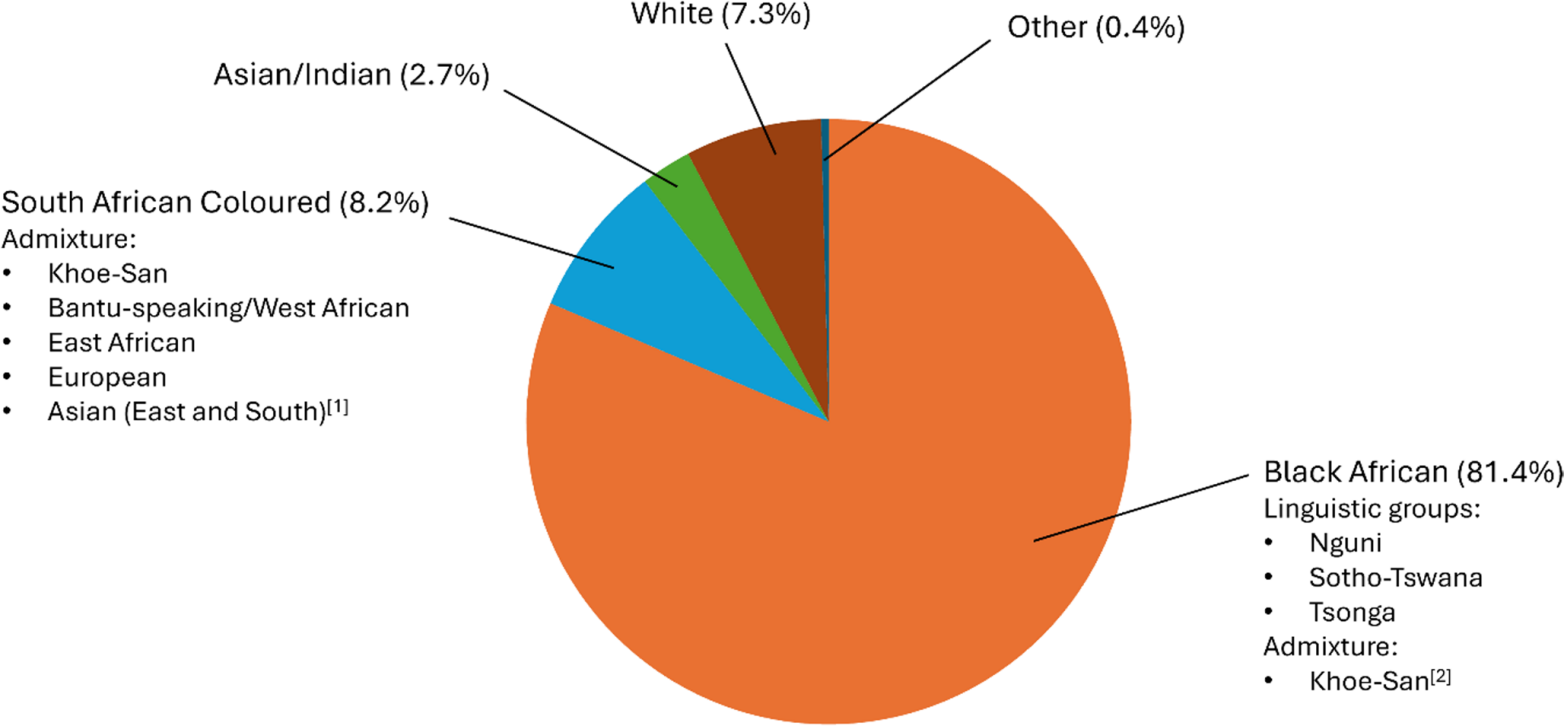
South Africa’s racial demographic composition, with ancestry admixture indicated. For Black Africans, there is fine-scale population structure across major linguistic groups. Data from the most recent (2022) census (https://census.statssa.gov.za/). [1] Lankheet et al. 2024; [2] Sengupta et al. 2021

In the Global North, various genetic services are available in service of personalised medicine, including genetic testing and counselling, which are invaluable tools for diagnostics, prognostics, and family planning (McPherson 2006). However, Africa’s participation in effective and accurate personalised medicine has remained limited, due to a dearth of African genomic data and poor infrastructure (Kamp et al. 2021) — many of the genetic tests that are currently available are not developed for an African context. A delayed understanding of African-specific causal, as well as protective, genetic variants has culminated in an inability to further translate new genetic information into comprehensive genetic tests. While this is being addressed by recent initiatives (Owolabi et al. 2023; Radouani et al. 2020), South Africa remains a unique case due to the ethnic diversity, particularly admixture, present in the country (Fig. 1). The development of appropriate diagnostic/prognostic genetic tests for the South African context requires increased investment in genetic testing. However, to justify increased investment, it is necessary to gauge what the supply and demand currently are for genetic services, particularly genetic testing.

There is a dual healthcare system, both public and private, within South Africa, with the majority of the population utilising public, government-funded healthcare (Mhlanga and Ndhlovu 2021). This is largely due to remarkably high economic inequalities within the South African population, with a Gini coefficient of 0.63 (World Bank 2018). These inequalities stem from apartheid, where non-Whites were completely segregated from the White population, including geographically and educationally (Clark and Worger 2022). Racial segregation began in 1910, worsening with the start of apartheid in 1948, which only fell out of practice in 1994 (Clark and Worger 2022). This has resulted in a unique scenario, where the majority (Fig. 1), rather than the minority, are marginalised. Within the healthcare system, there are severe inequalities between public (predominantly utilised by those that were segregated) and private healthcare that persist from apartheid (Coovadia et al. 2009; Mhlanga and Ndhlovu 2021), resulting in private healthcare receiving the majority of financial and human capital (Gomes et al. 2024).

Genetic services (specifically testing and screening) were formally integrated into the South African healthcare system in 1977 (Kromberg et al. 2013), with the need for genetic counselling services being acknowledged in 1988 (Wessels et al. 2024). Currently, common prenatal and newborn screening and testing is available across a broad range of institutions (including district and regional hospitals; National Department of Health [NDoH] 2021). Specialised pre/postnatal and post-early childhood genetic services have thus far been primarily restricted to tertiary state hospitals for those who cannot access private healthcare (Kromberg et al. 2013; Malherbe et al. 2017; NDoH 2021). Only two of the nine provinces have actively practising medical geneticists and genetic counsellors in the public sector (Gomes et al. 2024). This presents geographic limitations to genetic testing and counselling, as these hospitals are often inaccessible to rural communities (Kromberg et al. 2013; Malherbe et al. 2017). The availability of genetic tests in South Africa also differs per disease, making genetic investigations and confirmations of tentative diagnoses difficult (Wiener et al. 2023), particularly when considering the lack of non-European genomic data (Kamp et al. 2021) and the admixture and population structure present in South Africa (Lankheet et al. 2024; Sengupta et al. 2021). All of this results in these services being underutilised despite recognition of their importance (Gilfillan et al. 2025; Naidoo and Reddy 2022; Thom and Haw 2021; van Wyk et al. 2016).

Genetic counsellors and medical geneticists, both of whom facilitate patients’ comprehensive understanding of genetic information, are integral in the dissemination of genetic test results (Gomes et al. 2024; Greenberg et al. 2012). As of 2024, there were 13 practising medical geneticists in South Africa (9 in the public sector, 4 in the private sector), and 28 practising genetic counsellors (10 in the public sector, 18 in the private sector; Gomes et al. 2024). Considering the whole South African population, these practising medical professionals only serve 10% of the public sector (which over 70% of the population utilises; Stats 2025b) and 42% of the private sector. To reach full capacity, South Africa requires 500 additional genetic counsellors and 119 additional medical geneticists (Gomes et al. 2024). The appropriate implementation of clinical genetic testing requires some form of genetic counselling from trained personnel, as per South African guidelines (NDoH 2021). While genetic nurses were previously assigned in South Africa, when attention shifted to infectious diseases, posts for genetic nurses were reduced and many were reassigned or emigrated (Malherbe et al. 2017). Currently practising genetic counsellors in South Africa do believe, and hope, that genetic services (starting with genetic testing) will expand and become more broadly available across the country, largely attributing this to increased demand (Gilfillan et al. 2025).

To move towards improved accessibility, and accuracy, of genetic testing, it is necessary to investigate other potential barriers to utilisation. While financial and geographic barriers to uptake can be addressed at the governmental level, this would require a large resource investment, which should be preceded by ascertaining consumer need and demand as an indicator of potential uptake. Quantifying the need and demand is integral for informing policy and legislation — South Africa is currently struggling with a unique epidemiological state consisting of a high burden due to communicable diseases, an ever-increasing burden due to a rise in non-communicable diseases (NCDs; Wong et al. 2021), high rates of maternal and neonatal mortality (Damian et al. 2019), and high rates of interpersonal violence (Prinsloo et al. 2022). Rare diseases affect 4.8% of South Africans (Malherbe and Odendaal 2025), and congenital diseases affect 7% of yearly births (NDoH 2021). The largest disease burdens that South Africa faces are from communicable diseases (contributing 29% of years of life lost) and NCDs (contributing 20.7% of years of life lost; Neethling et al. 2020). Genetic testing would be particularly beneficial for these two major contributors, especially as the prevalence of NCDs continues to rise (Gouda et al. 2019). Communicable diseases and NCDs both have genetic risk factors which affect infection susceptibility for the former and the development of the latter (Olaniyan et al. 2024). Genetic testing availability and accessibility are likely to remain low if the existing infrastructure and policies continue to remain focused on communicable diseases; therefore, it is necessary to investigate any practical considerations surrounding the implementation of genetic testing, with the aim to tackle the burgeoning threat of NCDs. This narrative review identifies and discusses various sociocultural factors that negatively impact demand and uptake of genetic testing, and concludes by demonstrating how these factors can contribute to stigmatisation against genetic testing in South Africa.

## Methods

**Fig. 2:**
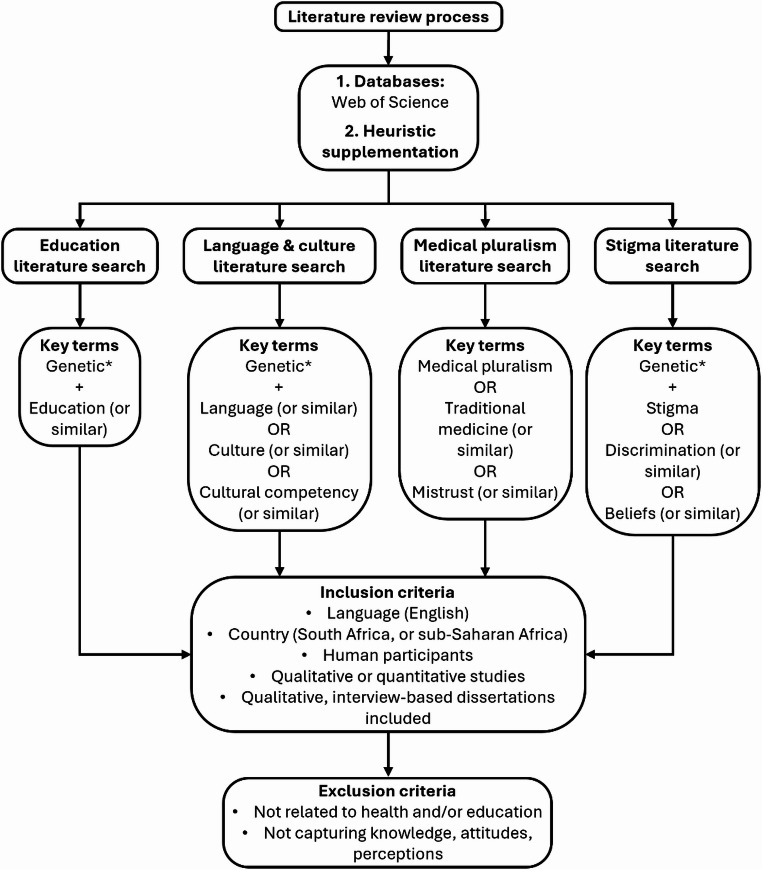
Flow chart illustrating the literature review identification and selection process used in this review

This narrative review utilised an iterative search strategy followed by a qualitative thematic synthesis of relevant literature. A systematic search was performed using combinations of free-text keywords that capture potential barriers to the uptake of and demand for genetic testing, as well as any stigmatisation. The generated keyword categories were based on Western literature regarding stigma and genetic testing. The literature identification and extraction process is depicted in Fig. 2. Due to the scarcity of recent research directly on the topic of genetic testing, stigma, and barriers to uptake in South Africa, we additionally identified useful literature heuristically to provide information not present in the articles identified via the systematic search.

### Education

Healthcare practitioners’ awareness of genetic testing and knowledge of genetics varies across sub-Saharan Africa and low- and middle-income countries (LMICs) in general (Walters et al. 2024; Zhong et al. 2021). Despite the importance of genetic services in South Africa being acknowledged in the 1970s (Kromberg et al. 2013; Wessels et al. 2024), primary healthcare providers (pHCPs) have low knowledge of genetics and express discomfort with discussing genetics with patients (Kroon et al. 2025; Walters et al. 2022). Even when knowledge of genetics and awareness of genetic testing are high, largely due to self-learning rather than offered training, pHCPs are often unaware of genetic testing being offered locally (Naidoo and Reddy 2022; Thom and Haw 2021), are unaware of referral procedures, or are unsure of which patients to refer (Thom and Haw 2021). pHCPs are generally viewed as the first port of call for information regarding genetic testing and subsequent referrals, which would necessitate knowledge of which diseases are genetically determined (Morris et al. 2015; van Wyk et al. 2016), as well as an understanding of follow-up care post testing (Walters et al. 2022).

Despite pHCPs’ low awareness of genetic testing, increasing interest in this service has been reported in South Africa (Gardiner et al. 2019; Morris et al. 2015; Naidoo and Reddy 2022). However, public awareness of this service appears to be low (Morris et al. 2015). Several studies note an incomplete understanding surrounding heritable traits (Matshabane et al. 2021; Morris et al. 2015; Penn et al. 2010; Solomon et al. 2012), and limited consideration of genetics being the causative agent underlying certain diseases (Gardiner et al. 2019; Matshabane et al. 2020, 2021; Penn et al. 2010). However, this appears to differ according to education attainment level (e.g., Naidoo and Reddy 2022). Effects of education on genetics knowledge are to be expected; for example, the foundational concepts of genetics are taught in the Life Sciences curriculum (Department of Basic Education 2011), which is an elective, rather than compulsory, subject. In addition, many South Africans in racially marginalised groups attend secondary schools that are under-resourced, understaffed, and often overcrowded, a lingering consequence of apartheid (Nkabinde 2016). Life Sciences teachers report a lack of institutional support and technological resources and a culturally insensitive curriculum, both of which severely hamper their ability to teach the subject (Chuene and Teane 2024; Ngwenya and Mavuru 2021; Ojo and Mathabathe 2021; Ramaila et al. 2025). This is exacerbated in rural areas, as smaller schools, which often have staff shortages, will appoint teachers who are not qualified to teach Life Sciences (Chuene and Teane 2024). An understanding of basic concepts of genetics is essential to correctly understand the function of genetic testing; consumers may otherwise have misconceptions surrounding what the results of a genetic test entail (i.e., “passing a test” or diagnostic and therapeutic misconceptions; Masiye et al. 2017) and may not fully grasp the practical value of genetic test results.

## Language and culture

Linguistic differences lead to communication barriers, impeding understanding. In total, there are 11 recognised spoken languages in South Africa, and the dominant language varies geographically (Stats 2025a). Within the Indigenous African languages, there is often a lack of established scientific terminology (Kromberg and Jenkins 1997; Mpe 2023; Shingwenyana 2023). While some terms already exist in these languages, there are still terms that need to be borrowed from English (e.g., “DNA”, “genes”, “genetic predisposition”; Mpe 2023; Shingwenyana 2023; Solomon et al. 2012), though participants have recommended words, explanations, or analogies that they would use to explain these concepts in their mother tongues (Mpe 2023; Shingwenyana 2023). In their review of genetic counselling in South Africa, Wessels et al. (2024) report that the first Black genetic counsellor graduated in 2018. According to the authors, diversity is increasing. However, there is often a mismatch in languages between healthcare providers and patients (Deumert 2010; Levin 2006a, b; Morris et al. 2015; Penn 2007), reflecting a lack of healthcare providers (including genetic counsellors) who are fluent in Indigenous African languages, particularly ones who are also knowledgeable about the cultural practices and beliefs of different South African groups (Levin 2006a, b; Schlemmer and Mash 2006). More recent investigations into patient experiences with genetic counselling focus on patients who have been counselled in English (e.g., Bayley et al. 2025; Scott et al. 2023), which limits investigations into the impact of linguistic mismatch. Language barriers result in a reliance on interpreters (Deumert 2010; Greenberg et al. 2012), who may not be qualified to inform the patient (Hunt and Swartz 2017; Penn 2007) and will need to be skilled enough to paraphrase the information according to each person’s unique lived experience. The breakdown of communication, either directly from genetic counsellors or pHCPs or indirectly through interpreters, results in necessary information being ineffectively communicated (Hunt and Swartz 2017; Solomon et al. 2012), leading to mistrust and distrust, misunderstandings, and dissatisfaction with biomedicine and biomedical professionals (Levin 2006b; Morris et al. 2015; Schlemmer and Mash 2006).

Due to historical (and ongoing) racial marginalisation and health disparities, there is substantial pre-existing mistrust and distrust in Western medicine and biomedical professionals, which has a negative impact on health behaviour (Prall 2024). Distrust stems from personal assumptions that specific healthcare providers may be incompetent or have ulterior motives, while mistrust is broader, aimed at the healthcare system as a whole, and is often rooted in historical and structural marginalisation (Griffith et al. 2021). The health-seeking behaviours of patients, their needs, and how they understand and perceive diseases are shaped by their cultural and religious backgrounds and general lived experiences (Bila and Carbonatto 2022; Dlamini et al. 2025). Cultural disconnect is prevalent, as Western medicine does not incorporate the multifactorial disease explanations that different cultural groups have (Merten et al. 2010). Rather than a causative genetic explanation, there is often a multifactorial explanation for the development of diseases, with all potential factors being considered concurrently and given different weightings of importance (Matshabane et al. 2021). These factors include social determinants of health (e.g., psychosocial factors, behaviour, and socioeconomic factors) and cultural explanations (Matshabane et al. 2020, 2021; Penn et al. 2010). Cultural explanations include supernatural factors influencing the development of disease, which is a commonly accepted explanation within the culturally diverse South African context (Egbe et al. 2014; Matshabane et al. 2020, 2021; Penn et al. 2010; Solomon et al. 2012). Many Indigenous African communities also have a fatalistic, collectivist culture (Jegede 2009; Kromberg and Jenkins 1997), which the Western model of medicine is not tailored towards (Zhong et al. 2021), as it has a distinct focus on diagnosis of the individual, with little consideration of family members or the community (Ramugondo et al. 2017). Therefore, the collectivist nature of these communities is particularly of interest with regards to uptake, as the benefits and risks of the results, and the general wellbeing of the patient, do not just impact the individual — they impact the immediate family, the extended family, and other community members (Jegede 2009; Merten et al. 2010). These multifactorial understandings of disease, combined with the lack of cultural competency of many South African medical professionals, often lead to patients pursuing alternative treatment and increasing resistance towards Western medicine.

## Medical pluralism

Other factors that contribute to medical mistrust and distrust in sub-Saharan Africa include long wait times, organisational inconsistency, medication stock-outs (Merten et al. 2010), and biomedical professionals discrediting patients’ different cultures and belief systems (Prall et al. 2025). Word-of-mouth is a strong driving force for health-seeking behaviours in South African communities, particularly those in more rural settings (James et al. 2018; Rutebemberwa et al. 2013; van der Watt et al. 2020). Negative experiences with genetic services will be divulged and spread, resulting in increased avoidance; conversely, positive experiences would result in increased uptake, as is seen with traditional healers (Rutebemberwa et al. 2013). Traditional healers are widely used, especially in rural communities, where medical services are sparse and not easily accessible (Batisai 2016; Ekpor et al. 2024; Hughes et al. 2021). Patients often seek out traditional medicine due to ease of availability, affordability, community influences (Batisai 2016; Hughes et al. 2021, 2022; James et al. 2018; Rutebemberwa et al. 2013), and as a last-ditch effort when Western medicine has failed (Batisai 2016). Additionally, considering that understandings of disease aetiology often include cultural and behavioural explanations, traditional healers may present a more attractive option, as the explanations they offer may align more closely with the patients’ belief systems (Ekpor et al. 2024; Hughes et al. 2021, 2022; James et al. 2018; Merten et al. 2010).

Traditional medicine may be the first choice for many individuals, rather than an alternative, due to patients’ causal explanations of their disease; if there is a pre-existing supernatural and/or cultural explanation, a traditional healer (who accepts and acknowledges ancestral and spiritual influences) would be a desirable choice (Bila and Carbonatto 2022; Egbe et al. 2014; Schierenbeck et al. 2018). However, many patients access and utilise traditional and Western medicine simultaneously (Hughes et al. 2022; Penn et al. 2010; Solomon et al. 2012) or in a sequential, zigzag pattern (Batisai 2016; Schierenbeck et al. 2018). The solitary use of traditional medicine could contribute to avoidance of genetic testing for two key reasons. First, if symptoms improve over the short-term through the use of traditional medicine, patients may delay seeking treatment from regulated medical practices and subsequent testing referrals (Kromberg and Jenkins 1997; Moshabela et al. 2016, 2017). Second, traditional healers will sometimes claim that they can “cure” various diseases, and patients believe this (Rutebemberwa et al. 2013). The results of genetic testing may contradict these beliefs (Kromberg and Jenkins 1997), so the perceived benefit of traditional medicine would outweigh that of genetic testing (Batisai 2016; Rutebemberwa et al. 2013), and, if trust and rapport with the providers of these services are already low, patients may elect to avoid testing completely.

A lack of trust and understanding also exists between traditional healers and biomedical healthcare practitioners (Wollie et al. 2025). Since many patients seek out traditional healers first, a mutual understanding is needed to foster cooperation (Esterhuizen et al. 2018). pHCPs may not actually be the first port of call for referrals, but rather a secondary one. If traditional healers lack trust in biomedical professionals, they may feel resistant to referring patients to them (Wollie et al. 2025). While traditional healers do refer patients when deemed necessary (van der Watt et al. 2020), there is no clear definition of what “necessary” entails, and what traditional healers’ awareness is of genetic testing and genetically determined diseases, necessitating a collaborative understanding between traditional healers and biomedical professionals.

## Stigma

Goffman defined stigma as “an attribute that is deeply discrediting” (Goffman 1963, p. 3). Later, Link and Phelan (2001) reconceptualised stigma, suggesting that it exists when there is a convergence of interrelated components: “when elements of ‘labelling’, stereotyping, separation, status loss, and discrimination co-occur in a power situation that allows the components of stigma to unfold” (p. 367). Stigma has been linked to poorer health outcomes and worse health- and treatment-seeking behaviour, which would include the uptake of genetic testing (Kane et al. 2019), and fundamentally causes health inequalities (Hatzenbuehler et al. 2013). There are several categories of stigma: internalised/self-stigma, which involves the stigma that one exerts on themself; associative stigma, which is experienced by those who care for or are associated with a stigmatised individual; experienced stigma, where the stigmatised individual experiences discrimination from others; and anticipated stigma, which refers to stigmatisation that is expected, but has not yet occurred (Stangl et al. 2019).

The sociocultural barriers discussed above can all contribute to the stigmatisation of genetic testing. Several studies have identified links between sociocultural factors and attitudes towards genetic testing (Table 1). How these factors affect motivation or interest in genetic testing varies across demographic factors (Angelo et al. 2024; Catz et al. 2005; Likhanov et al. 2023). However, a substantial proportion of these studies have been conducted in Western countries, with a large portion of studies focusing on racial/ethnic minority groups (who may already be Westernised) and being led by Western or Westernised authors. This fails to capture the nuance of potential stigma mechanisms and barriers in different contexts. South Africa is a notable system in which to investigate stigma, specifically with regards to its sociopolitical history and the diversity of those that live in the country. As stated by Ramugondo et al. (2017), “the definition of stigma is inextricably linked to power relations and structures”.

**Table 1:** Identified factors (from Western studies and studies identified in this review) that influence the uptake of genetic testing and could therefore act as potential sources of stigma

Potential factors influencing attitudes and uptake (South Africa)	Identified factors influencing attitudes and uptake (Western)	Sources
Education	Patients’ genetics knowledge, understanding, and awareness of services	Hann et al. 2017; Kinney et al. 2010; Likhanov et al. 2023
	Healthcare providers’ knowledge and referrals	Catz et al. 2005; Marzuillo et al. 2013; Sussner et al. 2014
Language and culture	Religiosity and cultural beliefs	Hann et al. 2017; Hesse-Biber et al. 2024; Kinney et al. 2010; Likhanov et al. 2023
	Familial or group perspectives	Hesse-Biber et al. 2024; Sussner et al. 2014
	Language barriers	Catz et al. 2005; Sussner et al. 2014
Medical pluralism	Medical mistrust and distrust	Angelo et al. 2024; Austin et al. 2025; Hesse-Biber et al. 2024; Sussner et al. 2014

In South Africa, considering the knock-on effects of apartheid (especially those relating to economic and education opportunities) and the diverse ethnic and cultural context, stigma takes on a distinct intersectional dimension. Intersectionality, first defined by Crenshaw (1989), refers to how different social and political identities (e.g., race, gender, socioeconomic status, disability/disease) intersect with one another and interlock with reinforcing systems of oppression, power, inequality, and social exclusion, leading to large disparities in quality of life (Metheny et al. 2025). While certain lived experiences may be similar across Black South Africans, for example, some experiences will differ across gender (and biological sex), sexuality, culture, age, and socioeconomic status. Viewing these factors in isolation, or at the population level, masks experienced inequities (Young et al. 2020). The concept of intersectional stigma draws on the ideas of intersectionality, specifically accounting for the convergence and compounded effect of having multiple stigmatised identities (Relf et al. 2021). In addition to being multidimensional, intersectional stigma is also multilevel — it operates at institutional, community, interpersonal, and individual levels, and considers how these levels interact to shape stigma (Earnshaw et al. 2022). Intersectional stigma can affect health-seeking behaviours, access to care, and healthcare satisfaction (Bradley et al. 2025; Butler et al. 2025; Foster et al. 2024). South African studies have primarily investigated intersectional in the context of communicable diseases (e.g., Bergman et al. 2022; Bradley et al. 2025; Foster et al. 2024); insights regarding intersectional stigma in the context of NCDs and genetic testing specifically have not yet been generated.

The stigmatisation of genetic testing can stem from stigma surrounding the diseases and disorders that are being tested for. Cultural and behavioural explanations will inevitably affect attitudes towards those who are affected and the stigma that is anticipated and experienced (Zhong et al. 2021). Generally, in LMICs, the anxiety surrounding genetic testing relates to fear of a positive result and the implications thereof (Naidoo and Reddy 2022; Zhong et al. 2021), which could be informed by the pre-existing stigma surrounding certain diseases and anticipated stigma that those being tested may be subjected to. However, the anticipated and experienced stigma resulting from this are intersectional. For example, there is often gendered blame towards mothers of affected children (de Vries et al. 2020; Penn et al. 2010; Zhong et al. 2021), so biological females may have different perceptions towards genetic testing in comparison to biological males.

When investigating intersectionality, a relevant concern is whether seemingly healthy individuals would be willing to accept the risk of adding an additional marginalised identity (depending on the pre-existing stigma of each disease) into their matrix of marginalisation through the process of genetic testing. This is something that needs to be clearly established when receiving informed consent. A patient’s health-seeking behaviour in a collectivist culture is strongly influenced by the perceptions of the community (Morris et al. 2015), as patients often seek external validation and advice before making decisions (Kromberg and Jenkins 1997; Merten et al. 2010). In this case, stigma cannot solely be studied from the perspective of the patient; instead, it must extend further into the perceptions of the community as a whole. In cases where biomedical professionals are not sufficiently culturally competent, a collective stigma surrounding genetic testing and Western medicine may be reinforced within the community, and result in patients altering their health-seeking behaviours (Prall et al. 2025), choosing to seek alternative treatments and advice regarding their diseases (James et al. 2018). Utilising genetic testing then not only puts patients at risk of stigmatisation depending on their genetic results (e.g., if it is revealed that they are predisposed to a disease), but could also reinforce the community’s negative perceptions of genetic testing. This could be worsened if the results of the testing (whether those be for diagnostics or therapeutics) conflict with existing belief systems or with opinions from traditional healers, especially if knowledge and understanding of basic genetic concepts remains limited. There may be concerns regarding increased stigma (especially internalised and associative stigma) with genetic testing, particularly discovering that a disease is genetically rather than environmentally determined (though this is not always the case; cf. Matshabane et al. 2020, 2021). Genetic testing could reduce certain types of stigmas. Several South African participants have noted that a genetic explanation would bring “peace of mind” and reduce self-blame (Gardiner et al. 2019; Matshabane et al. 2020). Participants have also reported that despite fears surrounding a positive test result, they were still interested in genetic testing, as the perceived benefits outweighed the costs (Naidoo and Reddy 2022).

A holistic and inclusive manner of addressing potential stigma surrounding genetic testing can be facilitated by understanding the underlying perceptions and stigma mechanisms in a South African context. However, this cannot be achieved if stigma has not yet been extensively characterised across different populations and intersections, especially for seemingly healthy people and those with multiple marginalised identities. In the context of apartheid and segregation, those who are currently marginalised, have limited access to well-resourced schools, have non-Western cultural beliefs, do not speak historically White languages, and utilise high levels of medical pluralism are in the vast majority. In order to effectively reduce any potential stigma surrounding genetic testing, it is necessary to have a complete understanding of the intersectional nature of stigma (Sievwright et al. 2022), which will need to encompass as many lived experiences as possible, especially ones that have been historically overlooked.

## Limitations and future research

**Fig. 3:**
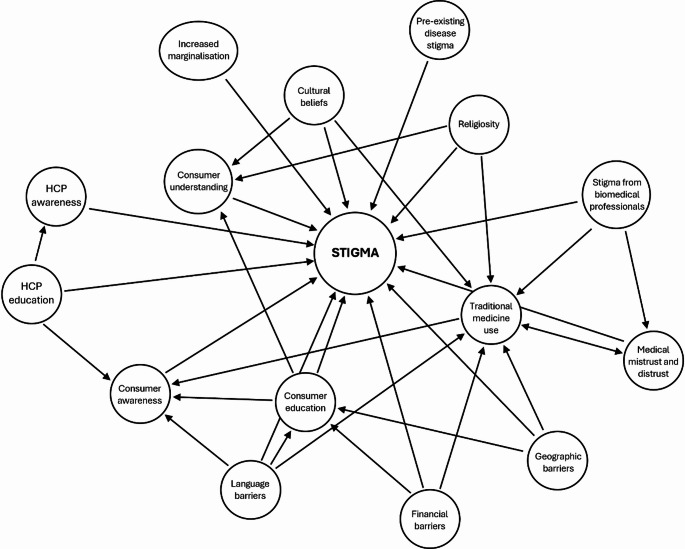
Theoretical directed acyclic graph depicting the potential causes of stigma in a South African setting and how these causes intersect. HCP = healthcare provider

This narrative review was limited by the paucity of South African studies that have explicitly investigated the barriers to uptake and utilisation of genetic testing, as well as studies that have worked towards quantifying demand. Due to this scarcity, this review is only able to suggest some potential barriers to uptake, and how these barriers may influence the stigmatisation of genetic testing and tested individuals. However, several avenues for future research can be identified from these knowledge gaps. As a means of conceptualising future research, Fig. 3 illustrates a theoretical causal network regarding genetic testing stigmatisation, considering the barriers identified in this review. Each node in Fig. 3, as well as their visualised intersections, could form the basis for future empirical research.

As mentioned, many studies have focused their investigations of individuals who are already diagnosed and have often been living with their diseases for extended periods of time. It would be worthwhile to gauge the stigma towards genetic testing from those who are currently asymptomatic or unaffected, especially those who are of a younger demographic, as they will be the ones to benefit most if there is future investment into genetic services. There may be generational differences in knowledge and stigma towards diseases (e.g., Mashini et al. 2024), the cultural/spiritual weighting given to disease aetiology (e.g., Digwamaje and Tadi 2024), and awareness of genetic services. This may also differ based on different population groups and their pre-existing cultural explanations for disease and the stigmatisation of different diseases. Considering the diverse cultural landscape of South Africa, evaluating the effect of different cultural explanations for disease on stigmatisation of genetic testing (and genetically determined diseases) would be of great benefit. In addition to this, multigenerational households are not uncommon in South Africa (Stats 2025b), which may influence how belief systems are passed down. Understanding and addressing stigma at the level of the individual, the familial unit, and the community is therefore necessary to allow patients to feel socially safe in accessing genetic services when required. A multitude of intersections exist in South Africa, which introduces many research questions that have yet to be asked. The unique situation of having a marginalised majority, along with the relatively recent end of apartheid, presents an interesting landscape to investigate intersectional stigma. Appropriate and effective intervention strategies to limit the stigmatisation of genetic testing will require understanding what underlies stigma, which populations experience this stigma, which stigma mechanisms are predominantly experienced, and how this manifests. This would be beneficial for tailoring awareness campaigns for genetic services with the goal of increasing uptake and awareness, and could inform teaching materials for biomedical professionals to improve cultural competency.

When determining the stigmatisation and knowledge of genetically determined diseases, genetic testing, and other genetic services, education should be explored in more depth. The South African studies mentioned in this review that investigate these perspectives either (1) broadly classify education without determining whether relevant subjects were taken (Egbe et al. 2014; Matshabane et al. 2021, 2022; Solomon et al. 2012) or (2) do not include demographic information on education (Gardiner et al. 2019; Penn et al. 2010). Therefore, it is unclear whether stigma and knowledge are affected by how genetics is taught during secondary and tertiary education, and whether the teaching practices of these topics should be modified. If this were investigated explicitly and it is found that participants who have taken relevant subjects have better understandings and reduced stigma, this may indicate that these concepts would be better placed into a compulsory subject, especially if the accessibility and availability of genetic services increases.

Aside from patients, further investigations into biomedical professionals’ awareness, knowledge, and opinions are warranted. Kroon et al. (2025) reported no improved uptake of genetic testing and genetic counselling in South Africa, despite apparent interest, a decade after physician needs were assessed and technologies were developed to bridge the implementation gap. As their survey was comprised of closed-ended questions, it is not clear what has resulted in this lack of uptake. While unclear referral procedures, a lack of confidence in genetics knowledge, and poor awareness of genetic testing have been noted (Kroon et al. 2025; Naidoo and Reddy 2022; Thom and Haw 2021), it may be beneficial to work with pHCPs to revisit existing materials and develop new materials to actionably increase clinical implementation. Assessing traditional healers’ knowledge and attitudes towards genetically determined diseases, genetic testing and referrals, and disease aetiologies would also be beneficial. This may allow for the creation of guidelines surrounding which diseases (and associated symptoms) would require referrals to biomedical professionals, fostering collaboration, cultural competency, and understanding.

## Conclusion

Considering the disease burdens that South Africa is currently experiencing, increased efforts towards preventative care are warranted, as preventative care is generally more cost effective than acute care (Probst-Hensch et al. 2011). However, before reallocating limited resources towards preventative care, specifically towards genetic services, the knowledge, attitudes, perceptions, and stigma towards genetic testing should be completely and holistically categorised to better understand whether there is a demand for these services and what can be done to increase uptake. Financial and geographic accessibility are factors that can be addressed at a governmental level; however, it would be unwise to address these barriers if it is unclear whether there is sufficient demand from consumers, as improving infrastructure would be time and resource intensive. We have discussed several sociocultural factors that influence demand and perceptions regarding genetic testing, specifically insufficient education for both patients and pHCPs, linguistic and cultural barriers to understanding and rapport, and the complex issue of medical pluralism and multifactorial understandings of disease aetiology. These likely contribute jointly towards a unique and intersectional stigmatisation of the use of genetic testing in South Africa. Currently, there is no clear understanding of the stigma mechanisms and the existing stigma that may act as hurdles towards complete preventative and personalised care in a South African context, though some potential mechanisms have been considered here (Fig. 3). It would be worthwhile to investigate consumers’, especially marginalised groups’, stigma surrounding genetic testing to elucidate feasibility, need, and demand. If need and demand are high, then it is possible to advocate for furthering the development of this service. This is a gap in knowledge that should be filled before we can work towards health equality for all South Africans and a decreased, and manageable, disease burden.

## Data Availability

All data are available upon reasonable request. The data generated herein are bibliographical in nature.
